# Treatment of depression and/or anxiety – outcomes of a randomised controlled trial of the tree theme method® versus regular occupational therapy

**DOI:** 10.1186/s40359-018-0237-0

**Published:** 2018-05-23

**Authors:** A. Birgitta Gunnarsson, Petra Wagman, Katarina Hedin, Carita Håkansson

**Affiliations:** 1 Department of Research and Development, Region Kronoberg, PO Box 1223, SE-351 12 Växjö, Sweden; 2 Department of Clinical Neuroscience and Rehabilitation, University of Gothenburg, Gothenburg, Sweden; 3 School of Health and Welfare, Department of Rehabilitation, Jönköping University, Jönköping, Sweden; 4 Futurum, Region Jönköping County and Department of Medical and Health Sciences, Linköping University, Linköping, Sweden; 5 Department of Clinical Sciences in Malmö, Family Medicine Lund University, Lund, Sweden; 6 Division of Occupational and Environmental Medicine, Lund University, Lund, Sweden

**Keywords:** Adults, Affective disorders, Art therapy, Intervention, Mental health

## Abstract

**Background:**

Depression and anxiety disorders are a major concern in western countries, and because these often have a negative affect on everyday life interventions based on activities in everyday life are needed. The Tree Theme Method® (TTM) is a client-centred occupational therapy intervention designed to increase the ability to cope with, and to enhance satisfaction with, everyday life, both at home and at work. The aim of this study was to compare the short term outcomes of the TTM intervention with regular occupational therapy treatment for people with depression and/or anxiety disorders.

**Methods:**

This randomised controlled trial included patients from three counties in Sweden. Men and women with depression and/or anxiety disorders, ages 18 to 65, were randomised to either TTM or regular occupational therapy. Assessment data were collected at baseline and the follow-up directly after completing the intervention. Non-parametric and parametric statistical methods were used.

**Results:**

The questionnaires were answered by 118 patients at baseline and by 107 patients after completing the intervention. No significant differences in short term outcomes were found between the groups. Both groups showed positive significant outcomes regarding almost all aspects of activities in everyday life, psychological symptoms, and health-related and intervention-related aspects.

**Conclusions:**

Despite the lack of differences between the groups, the positive outcomes regarding activities in everyday life, psychological symptoms, and health-related aspects after completing the intervention indicates the need for further research on the long-term perspective of TTM compared to regular occupational therapy.

**Trial registration:**

Clinical Trials.gov: NCT01980381; registered November 2013.

## Background

Depression and anxiety disorders are a major concern in western countries [1, 2], impacting on the individual, their family, and the individual’s role in society. In Sweden, the lifetime risk of being affected by depression is 36% for women and 23% for men. About 25% of the general population will be affected by anxiety disorders at some point in life [2, 3]. Symptoms like low mood and anhedonia, worried thoughts, and feelings of tension [4] might cause problems in the individual’s everyday life at home, at work, and during leisure time [2]. Engagement in everyday life is of importance for the individual’s health and well-being [5], and this means being involved in a variety of meaningful activities in everyday life [6], i.e. to have a satisfying everyday life. Even though the Swedish National Board of Health and Welfare recommends medication and Cognitive Behavioural Therapy (CBT) as treatments to decrease symptoms of depression and anxiety, they also state that a satisfying everyday life along with having a sufficient social network is just as important [1, 2, 7]. Despite this, there is a lack of interventions focusing on well-being in everyday life [8], even though there is some research based on well-being in relation to quality of life [9]. This highlights the need for interventions, and evidence for these, that support the individual by enhancing well-being related to activities in everyday life in terms of performance of, and satisfaction with performance of activities in relation to self-care, productivity at home and at work, leisure, and social relationships.

It is common that people suffering from depression also have symptoms of anxiety and vice versa [10, 11]. Randomised controlled trials (RCTs) of patients with depression and anxiety disorders being treated with internet cognitive behavioural therapy (iCBT) [12] showed that iCBT compared to a waiting list was more effective regarding psychological symptoms and functional impairment. However, no RCT focusing on well-being related to activities in everyday life in those with depression and/or anxiety disorders has been identified. There are RCTs from the Netherlands [13, 14] comparing sick-listed employees with major depression receiving treatment as usual to those receiving treatment as usual along with adjuvant occupational therapy. Furthermore, a Swedish case-control study [15] on employed and sick-listed women with stress-related disorders was evaluated in terms of stress, depression, and anxiety. However, these three latter studies were based on occupational therapy, with the primary outcome of returning to work [13–15]. Thus, we have not found any RCT for people with depression and/or anxiety disorders aiming to increase the ability to cope with and to enhance satisfaction with activities in everyday life at home and at work.

The Tree Theme Method® (TTM) [16] is designed for a client-centred occupational therapy context, and it aims to increase the ability to cope with, and to enhance satisfaction with everyday life for people with depression and/or anxiety disorders. The TTM is based on art therapy [17] and life story telling [18], i.e. occupational storytelling and occupational story making, with a focus on activities in everyday life [5]. When the patient tells their life story it is also a way to reflect how to act and explain why they act in a specific way in a specific situation and context. This will lead to a possibility to experience life in coherence and to master everyday life. The TTM has previously been evaluated with a focus on processes. In a group of patients with depression and/or anxiety disorders, the intervention showed positive significant changes in everyday life, psychological symptoms, and health-related aspects [19, 20], and both patients [16, 21] and therapists [22] were satisfied with the intervention. The fact that the TTM intervention [19] showed that the therapeutic alliance was linked to treatment outcomes, is in line with systematic reviews [23, 24] showing that it is not only the chosen intervention that determines the outcomes of an intervention, but also the therapeutic relationship and how satisfied patients are with their treatment [23, 24].

Even though the TTM intervention has been evaluated with focus on processes, this occupational therapy intervention has not yet been evaluated compared to other treatments. This, together with the lack of RCTs based on occupational therapy for the target group, emphasise the need for evaluating the outcomes of the TTM compared to regular occupational therapy methods.

### Aims

The aim of this study was to compare the TTM intervention with regular occupational therapy regarding activities in everyday life, psychological symptoms of depression and anxiety, and health-related and intervention-related aspects before and after the intervention in people with depression and/or anxiety disorders.

## Methods

### Study design

This study was a prospective RCT in which 118 patients were randomised to the TTM intervention or to regular occupational therapy, i.e. a parallel group design. This study has been previously described regarding material and methods in a study protocol [25]. The paper follows the CONSORT guidelines [26], and the study was registered as Clinical Trial NCT01980381. This paper concerns data collected at baseline and the follow-up directly after completing the intervention.

### Study population

The study was conducted at primary health-care centres and general outpatient mental healthcare units in three counties in Sweden. Men and women with depression and/or anxiety disorders aged 18–65 years and reporting problems with their everyday life were invited to take part. Exclusion criteria were severe somatic illness or psychosis and/or difficulties understanding and filling out self-rating questionnaires. Occupational therapists who were specially trained in the specific frames and techniques of the TTM intervention recruited suitable participants to the study and performed both the TTM intervention and regular occupational therapy. The special training is described in detail in the study protocol [25].

### Interventions

The TTM intervention, which involves five sessions, and the regular occupational therapy aimed to increase the ability to cope with everyday life and to enhance satisfaction with everyday life. In order to compare the TTM intervention with regular occupational therapy, i.e. best practice, both interventions involved five sessions, even though regular occupational therapy is normally not structured within specific time limits.

### The TTM intervention

In the TTM intervention [16, 25], the patient told their life story with a focus on activities in everyday life. In each session, there was also a reflective dialogue between the patient and the occupational therapist. Each session started with progressive relaxation, followed by the patient painting trees representing different periods in their life. In the first session, the tree represented the present life situation, in the second session it represented childhood, and in the third it represented adulthood. At each session, the patient also identified tasks linked to their difficulties and needs in everyday life that they should complete prior to the next session. At the fifth and final session, based on the previous tree paintings and life story telling, the patient painted a tree representing the future. This was followed by a dialogue in which the patient made plans for their future by identifying the need for change and how to incorporate these changes into their everyday life.

### Regular occupational therapy

Each therapist defined what they meant by regular occupational therapy, i.e. best practice, to ensure that the treatment did not resemble the TTM intervention. Examples of such treatment could be dialogues and activities to maintain activities and structure in everyday life, learning to prioritise among various activities, prescription of assistive devices for cognitive impairments, and creative activities in order to handle anxiety and stress.

### Primary outcomes

Primary outcomes were activities in everyday life and psychological symptoms.

Activities in everyday life were assessed from the perspectives of 1) performance of activities and satisfaction with the performance of activities in everyday life, as measured by the Canadian Occupational Performance Measurement (COPM) [27]; 2) satisfaction with activities in everyday life, as measured by the Satisfaction with Daily Occupations (SDO) [28]; and 3) balance between activities in everyday life, as measured by the Occupational Balance Questionnaire (OBQ) [29]. The Swedish version of the COPM has been validated and shows high responsiveness to change [30] and clinical utility [31]. The Swedish version of the SDO has been validated [28, 32, 33], and it had adequate internal consistency in this study (Baseline: Cronbach’s α = 0.78; Follow-up: Cronbach’s α = 0.84). The Swedish version of the OBQ has been validated [29], and it had adequate internal consistency in this study (Baseline: Cronbach’s α = 0.87; Follow-up: Cronbach’s α = 0.92).

Psychological symptoms were measured by the Symptom Checklist-90-R (SCL-90-R) [34, 35]. The SCL-90-R is divided into nine subscales, and for the present study only the subscales for Depression and Anxiety were used. Further, the SCL-90-R also consists of three indexes – the Global Symptom Index (GSI), the Positive Symptoms Index, and the Positive Symptoms Total Index – all of which were calculated in this study.

Depression was assessed using two measures; the Montgomery-Åsberg Depression Rating Scale (MADRS-S) [36] and the Hospital Anxiety and Depression Scale (HADS) [37, 38]. The MADRS measures symptoms of depression in terms of specificity [39], and is developed to measure an individual’s scores at baseline and at follow-up, ie. to follow an individual longitudinally. Scores ≤12 were classified as no depression, scores from 12 to 19 were classified as mild depression, scores from 20 to 34 were classified as moderate depression, and scores ≥35 were classified as severe depression. Depression and anxiety were assessed with the HADS [37, 38]. The HADS measures general distress [39], i.e. general mental health, such as anxiety/distress as experienced in the individual’s body in general. The HADS consists of an Anxiety subscale (HADS-A) and a Depression subscale (HADS-D). Scores ≤6 indicated no state of anxiety or depression, scores from 7 to 10 indicated possible anxiety or depression, and scores ≥11 indicated probable severe anxiety or depression.

The Swedish version of the SCL-90 has found to be reliable and valid [40]. The Swedish version of the MADRS has been validated [41], and it had adequate internal consistency in this study (Baseline: Cronbach’s α = 0.85; Follow-up: Cronbach’s α = 0.91). The Swedish version of the HADS has been validated [42], and it had adequate internal consistency in this study (Baseline: Cronbach’s α = 0.87; Follow-up: Cronbach’s α = 0.91), as well the HADS-A (Baseline: Cronbach’s α = 0.80; Follow-up: Cronbach’s α = 0.84) and HADS-D (Baseline: Cronbach’s α = 0.85; Follow-up: Cronbach’s α = 0.90). All instruments, including their psychometric properties, are described in detail in the study protocol [25].

### Secondary outcomes

Secondary outcomes were various health-related and intervention-related aspects. Health-related aspects involved 1) sense of coherence, measured by the Sense of Coherence Scale (SOC) [43]; 2) experience of control, measured by the Mastery Scale [44, 45]; and 3) quality of life, measured by the Manchester Short Assessment of quality of life (MANSA) [46]. Intervention-related aspects involved therapeutic alliance and patient satisfaction. The therapeutic alliance was measured by the Helping Alliance questionnaire (HAq-II) [47], and patient satisfaction was measured by the Client Satisfaction Questionnaire (CSQ) [48] that the patients answered on their own after the intervention. The Swedish version of the SOC has been validated [49], and it had adequate internal consistency in this study (Baseline: Cronbach’s α = 0.81; Follow-up: Cronbach’s α = 0.82). The Swedish version of the Mastery has been validated [50], and it had good internal consistency in this study (Baseline: Cronbach’s α = 0.65; Follow-up: Cronbach’s α = 0.83). The Swedish version of the MANSA has been validated [51], and it had adequate internal consistency in this study (Baseline: Cronbach’s α = 0.77; Follow-up: Cronbach’s α = 0.84). The Swedish version of the HAq-II has been validated regarding its internal consistency (Cronbach’s α = 0.88 for the therapist version and Cronbach’s α = 0.91 for the patient version) [52], and it had adequate internal consistency in this study regarding the therapists’ version (Baseline: Cronbach’s α = 0.82; Follow-up: Cronbach’s α = 0.92) as well the patients’ version (Baseline: Cronbach’s α = 0.89; Follow-up: Cronbach’s α = 0.86). The Swedish version of the CSQ has been validated for its internal consistency (Cronbach’s α = 0.94) [53], and it had adequate internal consistency in this study (Follow-up: Cronbach’s α = 0.94). All instruments, including their psychometric properties, are described in detail in the study protocol [25].

### Power calculation and randomisation

The power calculation indicated 60 patients in each group to obtain an effect size of 3.6 for the outcome variable SDO (scale 9–63), with 80% power (*p* ≤ 0.05). A total of at least 120 patients should be included, and in order to compensate for possible dropouts we calculated for 130 patients. The randomisation was performed by an administrator. In order to ensure that each arm contained equal numbers of patients a blocked randomisation [54] was used, in which each group consisted of 20 envelopes, 10 for the TTM intervention and 10 for occupational therapy as usual. The trial was single blinded, i.e. that the allocation was blinded to the patient, and their therapist, until after the first data collection was performed. Furthermore, the allocated intervention was blinded to the project assistants who performed the data collection during the whole process. In total, 121 patients were recruited.

### Statistical analysis

The analyses were conducted using IBM SPSS Statistics 23.0 following the intention-to-treat-principle [55]. Data quality was checked and validated. There were some random missing data at baseline and follow-up. Missing data were handled as missing, and no imputations were made. Chi-squared tests were used to compare categorical data, and *t*-tests were used to compare mean ages at baseline (Table 1). Non-parametric methods [56] were used to compare data on ordinal scales – the Wilcoxon signed-rank test was used to analyse within-group effects, and the Mann–Whitney U-test was used to compare effects between groups (Table 2).

**Table 1:** Comparison of baseline characteristics for the patients (*n* = 118)

	TTM (*n* = 62) n (%)	Occupational therapy as usual (*n* = 56) n (%)	*p*-value (chi-squared test)
Gender:			0.80
Men	10 (16.1)	10 (17.9)	
Women	52 (83.9)	46 (82.1)	
Living status:			0.37
Single	19 (30.6)	24 (42.9)	
With someone	43 (69.4)	32 (57.1)	
Have children ≤18 years	23 (37.1)	18 (32.1)	0.77
Educational level:			0.63
University	20 (32.3)	15 (26.8)	
High school degree	31 (50.0)	27 (48.2)	
Elementary school	9 (14.5)	13 (23.2)	
No elementary school	2 (3.2)	1 (1.8)	
Main support:			0.62
Employed/Student	23 (37.1)	16 (28.6)	
Unemployed	4 (6.5)	6 (10.7)	
Others (parental leave, retired)	1 (1.6)	1 (1.8)	
Sick-leave (including 3 who work trained)	34 (54.8)	33 (58.9)	
Primary diagnosis:			0.44
Affective disorders (F31–38)	40 (64.5)	40 (71.4)	
Anxiety/obsessive disorders (F40–49)	22 (35.5)	16 (28.6)	
Medication:			
Insomnia	25 (40.3)	29 (51.8)	0.32
Depressive symptoms	43 (69.3)	41 (73.2)	0.45
Anxiety symptoms	19 (30.6)	23 (41.1)	0.48
Others (e.g. Antipsychotics, Central stimulants)	12 (19.4)	10 (17.9)	0.84

**Table 2:** Changes between measurements made before and after the intervention

	The TTM group				The other occupational therapy group				Comparisons of changes between groups
	Baseline Median (IQR)	After completing the intervention Median (IQR)	Change Median (IQR)	*p*-value	Baseline Median (IQR)	After completing the intervention Median (IQR)	Change Median (IQR)	*P*-value	*p*-value
Primary outcomes									
*COPM*									
Performance	4(3, 5)	5 (3, 6)	0 (0,1)	≤ .01	3 (3,5)	5 (3,6)	0 (1,2)	≤ .01	.60
Satisfaction	2 (2, 4)	4 (2, 6)	1 (0, 2)	≤ .01	3 (2, 4)	5 (3, 6)	1 (0, 2)	≤ .01	.59
*SDO*									
Activity level	7 (6, 8)	7 (6, 9)	0 (−1, 1)	≤ .01	8 (6, 9)	8 (6, 10)	0 (−1, 2)	≤ .01	.65
Satisfaction score	63 (50, 76)	65 (53, 77)	3 (− 8, 11)	.21	62 (53, 69)	64 (57, 72)	5 (−5, 11)	.02	.34
*OBQ*	23 (17, 30)	30 (21, 38)	5 (0, 12)	≤ .01	22 (15, 30)	30 (21, 37)	6 (2, 9)	≤ .01	1.00
*SCL-90-R symptom scales*									
Depression	83 (71, 91)	73 (58, 87)	−8 (−13, 2)	≤ .01	81 (70, 91)	71 (57, 90)	−2 (−15, 3)	≤ .01	.34
Anxiety	81(68, 93)	71 (53, 91)	−7 (−14, 4)	≤ .01	82 (67, 100)	78 (61, 93)	−7 (−16, 5)	.01	.73
*SCL-90-R indexes*									
GSI	81 (69, 96)	72 (59, 89)	−6 (− 14, 2)	≤ .01	79 (68, 95)	73 (58, 90)	−4 (−13, 2)	≤ .01	.74
Positive symptoms	71 (65, 86)	65 (56, 78)		≤ .01	70 (63, 77)	67 (55, 74)		≤ .01	.11
Positive symptoms total	70 (63, 76)	66 (56, 76)		.01	72 (65, 79)	69 (61, 76)		≤ .01	.27
*MADRS-S*	25 (20, 30)	20 (11, 29)	−3 (−8, 1)	≤ .01	25 (20, 32)	20 (14, 27)	−4 (−10, 0)	≤ .01	.46
*HADS*									
HADS-A	14 (10, 17)	12 (8, 15)	−2 (−5, 1)	≤ .01	14 (11, 16)	12 (9, 15)	−1 (−4, 1)	≤ .01	.63
HADS-D	10 (7, 13)	8 (3, 12)	−2 (−4, 0)	≤ .01	10 (7, 14)	7 (5, 11)	−2 (−4, 0)	≤ .01	1.00
Secondary outcomes									
*SOC*	47 (39, 55)	50 (44, 62)	5 (−1, 11)	≤ .01	46 (41, 57)	52 (46, 59)	2 (−4, 9)	.04	.23
*Mastery*	18 (16, 20)	18 (16, 22)	0 (−1, 2)	.03	17 (16, 19)	18 (16, 21)	1 (−2, 3)	.09	.94
*MANSA*	47 (37, 52)	50 (38, 61)	2 (−2, 6)	.02	45 (35, 52)	50 (39, 55)	3 (−1, 8)	.01	.75
*HAq-II*									
HAq-II OTs’	86 (80, 92)	96 (90, 106)	11 (7, 16)	≤ .01	85 (80, 90)	94 (90, 102)	9 (6, 15)	≤ .01	.27
HAq-II patients’	99 (93, 105)	106 (99, 111)	4 (−1, 10)	≤ .01	95 (91, 101)	102 (94, 107)	4 (0, 11)	≤ .01	.99
*CSQ*		26 (23, 30)				26 (24, 30)			.96

The IQR (interquartile range) shows the difference between the 25th and 75th percentiles, that is, it contains the central 50% of the observations. TTM, Tree Theme Method; COPM, Canadian Occupational Performance Measure; SDO, Satisfaction with Daily Occupations; OBQ, Occupational Balance Questionnaire; SCL-90-R, Symptom Checklist-90-R; GSI, Global Symptom Index; MADRS-S, Montgomery-Åsberg Depression Rating Scale; HADS, Hospital Anxiety and Depression Scale divided into an Anxiety subscale (HADS-A) and a Depression subscale (HADS-D); SOC, Sense of Coherence measure; MANSA, Manchester Short Assessment of quality of life; HAq-II, Helping Alliance questionnaire; CSQ, Client Satisfaction Questionnaire

## Results

**Fig. 1:**
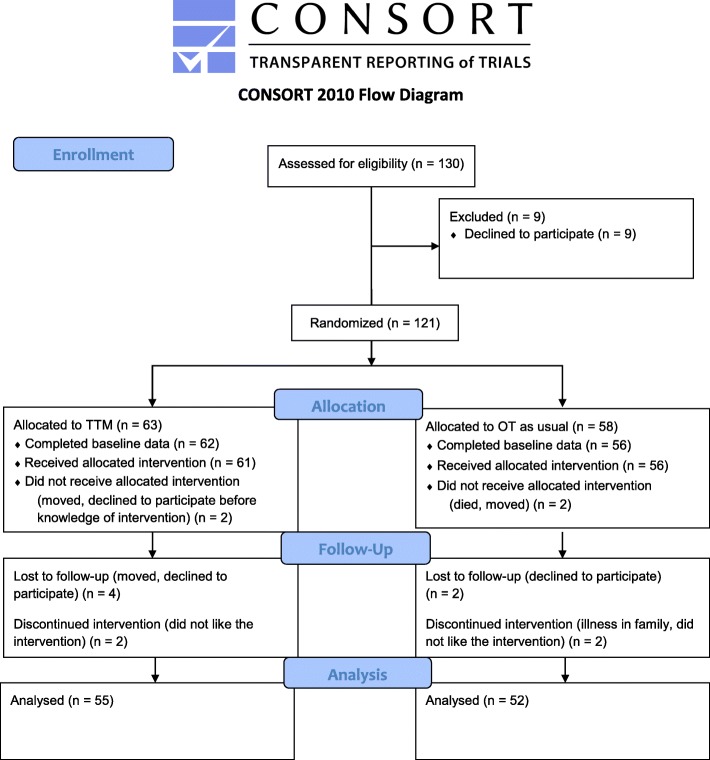
CONSORT 2010 Flow Diagram

Of the 121 recruited patients, 118 patients completed baseline data and 107 of them completed follow-up data (Fig. 1). There were no significant differences between the TTM intervention group and the regular occupational therapy group at baseline. The mean age of the patients in the TTM intervention group was 43.0 years (SD = 11.3, Range 19–63 years), and the mean age of the regular occupational therapy group was 40.1 years (SD = 12.6, Range 20–64 years) (*t*(116) = 1.46, *p* = 0.15). Other baseline characteristics are shown in Table 1.

No significant differences between the groups for the primary outcomes of activities in everyday life and psychological symptoms were identified at the follow-up. Both groups reported significantly higher ratings on all outcomes, except for the satisfaction with activities in everyday life (measured by the SDO), which did not show significant changes in the TTM intervention group (Table 2). For the secondary outcomes, i.e. various health-related and intervention-related aspects, no differences between the groups were found. However, here also the analysis showed positive significant outcomes in both groups, except for experience of control (as measured by the Mastery Scale), which did not show any significant changes in the regular occupational therapy group (Table 2).

## Discussion

### Study limitations

The main findings from this RCT are that no significant differences were seen between the TTM intervention group and the regular occupational therapy group in terms of activities in everyday life, psychological symptoms, health-related aspects, or intervention-related aspects. At the time of follow up, both groups showed improvements in all of these aspects.

A strength of the present study was that the study compared the TTM intervention with treatment as usual, i.e. regular occupational therapy, which is in line with Snappin [57], who argues that therapy as usual is a better alternative than placebo in clinical contexts [57]. Otherwise, it would be ideal to evaluate any effects of an intervention by comparing the intervention with a control based on a waiting list or an attention-placebo group [55]. Although, according to Snappin, there can be an ethical dilemma when offering an attention-placebo group to patients suffering from diseases [57] such as depression and anxiety disorders.

Another strength of the present study was the knowledge of the content of the regular occupational therapy treatment when comparing it with the TTM intervention. In general, regular occupational therapy is not structured to time and content, but in the present study the occupational therapists had limited time frames and had to decide upon and describe the content before the study started. On the other hand, in this case the regular occupational therapy treatment could also be seen as a limitation because it might have been more focused than when occupational therapy as usual is performed in a clinical context. Thus, in retrospect we could have performed the trial with an intervention group and a control group, and not with a parallel group design.

A further strength was the internal validity [55] as measured by baseline characteristics with no significant differences between the groups. Handling missing data is a challenge in RCTs [58], and because there was a lack of guidance on how to handle missing data in relation to each chosen measurement, we analysed the data both with and without imputed values, and we found no differences concerning any of the outcomes. This showed the robustness of the data and further strengthened the internal validity of the study [55]. We therefore decided to present the results with no imputed data. A weakness and perhaps a threat to internal validity measured by baseline characteristics with no significant differences between the groups [55] was that the same occupational therapists carried out the TTM intervention as well as the regular occupational therapy, and thus there was a risk for overlapping of the different treatments. To handle this, and to ensure that the occupational therapists followed the allocated intervention, a standardised protocol was developed for this study. After each completed intervention the therapists reported the content in each session, as a form of integrity check [59] to assess the adherence to the TTM procedures.

Also, we did not find any differences regarding the therapeutic relationship or any of the other outcomes between the different occupational therapy interventions that were used.

A final strength was that we handled threats to external validity [55] through the purposeful sampling and the choice of context. Therefore, this robust study might be generalised to other similar groups of patients and contexts. The fact that there were mainly female patients participating in this study might be seen as a weakness, indicating that the outcomes cannot be generalised to men. However, this is in accordance with previous research [10, 11] which showed that depression and anxiety disorders are more common among women than men, even though the distribution in this study was more skew than expected.

### Discussion of the outcomes

There were no differences in the primary outcomes between the groups, but the importance of this study is demonstrated by the improvements in both groups for performance of activities and satisfaction with performance of activities, as measured by the COPM. This has been found to be of clinical utility in helping patients to define realistic and desirable goals [60]. We can assume that the participants in the present study identified activities that were important in their everyday life that they wanted to change during the treatment sessions. This could be one explanation for the consistent improvements regarding various activities in everyday life in the TTM intervention as well as the regular occupational therapy treatment. Also, the rated balance between activities in everyday life showed improvements during the present study. Even though the values in the present study were lower at baseline and at follow up for both groups compared to previous research on general populations [61, 62], the balance between activities in everyday life is related to health and well-being [63]. Therefore, the positive outcomes regarding activities in everyday life might indicate that interventions focusing on activities in everyday life have a positive impact on everyday life and health. However, the improvements in both groups might also be due to the attention [55] you get when participating in a research study, and improvements in both intervention and control groups have been seen in previous research [13–15].

In the present study both groups decreased their ratings for psychological symptoms, which is in line with Eklund [64], in whose study an intervention focusing on everyday life also showed decreased symptoms of anxiety and depression. The importance of reducing symptoms of depression and anxiety are highlighted in the Swedish national guidelines for depression and anxiety disorders [1, 2], and various methods of psychological treatments are recommended. A study [12] from primary care found that psychological treatment such as iCBT leads to decreased psychological symptoms, although the impact of iCBT in everyday life was not fully evaluated. Studies have also shown a lack of evidence for CBT, such as Ekeblad et al. [65], who found no significant improvements in psychological symptoms when comparing iCBT and CBT.

There were positive outcomes in both groups, but there were two differences in outcomes between the groups. Firstly, there were no significant improvements in satisfaction related to everyday life, as measured by the SDO [28], in the TTM intervention group. An explanation might be that there were some more patients in the TTM intervention group, in comparison to the regular occupational therapy group, who scored their satisfaction in everyday life as lower at the time for follow up, compared to their scorings at baseline. Secondly, there were no significant improvements in experience of control, as measured by the Mastery Scale [44], in the regular occupational therapy group. An explanation might be the same, i.e. that there were some more patients in the other occupational group, in comparison to the TTM intervention group who scored their experience of control as lower at the time for follow up, compared to their scorings at baseline. One explanation to this might be the patients’ awareness of how their everyday life looks like, and is perceived. Maybe an individual realise the need for changes in everyday life, and maybe they have started with these changes, but to make changes persisted over time and the results of them takes longer time. Further follow-ups from this trial can give further knowledge regarding the outcomes.

Thus, to sum up, there are various possible methods for treating people with depression and/or anxiety disorders, and even though CBT is commonly recommended [1, 2] there is no single intervention that fits everyone. Therefore, to be client-centred [5, 66] there is a need for further development of various kinds of treatments like the TTM intervention. To obtain greater knowledge of the impact of various treatments, further studies comparing the effects of different occupational therapy treatments are needed. Also, longitudinal studies are needed in order to further evaluate whether the results from the present study will be stable over time or not.

### Clinical implications

Even though there were no differences in effects between the TTM intervention and regular occupational therapy treatment, there were positive improvements in both groups. This can indicate that occupational therapy treatments can be useful and valuable in increasing the ability to cope with, and to enhance satisfaction with, everyday life. However, at short-term follow up, the TTM intervention was on par with best practice for people with depression and/or anxiety disorders.

## Conclusion

In summary, the present study showed no significant differences between those who received the TTM intervention and those who received regular occupational therapy treatment. At the time for follow up after completing the intervention, both groups showed improvements, i.e. positive significant outcomes regarding activities in everyday life, psychological symptoms, health-related aspects, and intervention-related aspects. Thus, at least for the follow-up period in this study, the TTM intervention was on par with best practice. Despite the lack of differences between the groups, the positive outcomes after completing the intervention indicate the need for further research on the long-term perspective of the TTM intervention compared to regular occupational therapy treatments.

## Abbreviations

CBT: Cognitive Behavioural Therapy
CONSORT: Consolidated Standards of Reporting Trials
COPM: Canadian Occupational Performance Measurement
CSQ: Client Satisfaction Questionnaire
GSI: Global Symptom Index
HADS: Hospital Anxiety and Depression Scale
HADS-A: Anxiety subscale
HADS-D: Depression subscale
HAq-II: Helping Alliance Questionnaire
iCBT: internet Cognitive Behavioural Therapy
MADRS-S: Montgomery-Åsberg Depression Rating Scale
MANSA: Manchester Short Assessment of quality of life
OBQ: Occupational Balance Questionnaire
RCT: randomised controlled trial
SCL-90-R: Symptom Checklist-90-R
SDO: Satisfaction with Daily Occupations
SOC: Sense of Coherence
TTM: Tree Theme Method®
